# Intrathecal Resiniferatoxin Modulates TRPV1 in DRG Neurons and Reduces TNF-Induced Pain-Related Behavior

**DOI:** 10.1155/2017/2786427

**Published:** 2017-08-02

**Authors:** M. Leo, M. Schulte, L.-I. Schmitt, M. Schäfers, C. Kleinschnitz, T. Hagenacker

**Affiliations:** Department of Neurology, University of Duisburg-Essen, Hufelandstr. 55, 45122 Essen, Germany

## Abstract

Transient receptor potential vanilloid-1 (TRPV1) is a nonselective cation channel, predominantly expressed in sensory neurons. TRPV1 is known to play an important role in the pathogenesis of inflammatory and neuropathic pain states. Previous studies suggest interactions between tumor necrosis factor- (TNF-) alpha and TRPV1, resulting in a modulation of ion channel function and protein expression in sensory neurons. We examined the effect of intrathecal administration of the ultrapotent TRPV1 agonist resiniferatoxin (RTX) on TNF-induced pain-associated behavior of rats using von Frey and hot plate behavioral testing. Intrathecal injection of TNF induces mechanical allodynia (2 and 20 ng/kg) and thermal hyperalgesia (200 ng) 24 h after administration. The additional intrathecal administration of RTX (1.9 *μ*g/kg) alleviates TNF-induced mechanical allodynia and thermal hyperalgesia 24 h after injection. In addition, TNF increases the TRPV1 protein level and number of TRPV1-expressing neurons. Both effects could be abolished by the administration of RTX. These results suggest that the involvement of TRPV1 in TNF-induced pain offers new TRPV1-based experimental therapeutic approaches and demonstrates the analgesic potential of RTX in inflammatory pain diseases.

## 1. Introduction

Tumor necrosis factor- (TNF-) *α* is a proinflammatory cytokine that is expressed by a variety of cell types, including immune and neuronal cells. TNF is expressed by microglial cells and astrocytes in the central nervous system and by Schwann cells in the peripheral nervous system, especially after nerve injury [1]. In the development and maintenance of inflammatory and neuropathic pain, TNF effects are mediated by two distinct receptors, TNF receptor-1 (TNFR1) and TNF receptor-2 (TNFR2) [2]. Both TNF and its receptors are expressed in dorsal root ganglion (DRG) neurons and are upregulated after nerve injury [3, 4]. While numerous studies have shown a critical role of TNF in neuropathic pain, the exact mechanisms by which TNF elicits neuropathic pain are not fully understood. Intraganglional application of TNF induces pain-related behavior in rats, which is accompanied by mechanical and thermal hyperalgesia [5] and can be prevented by the preemptive use of TNF-neutralizing agents or by the inhibition of the TNF signaling pathway [6–8]. In addition to its influence on different ion channels such as voltage-gated sodium channels (VGSC) [9–11], TNF has been shown to sensitize the transient receptor potential vanilloid 1 (TRPV1), suggesting its crucial role for thermal hyperalgesia [9–11].

TRPV1 is a nonselective cation channel that is predominantly expressed in sensory neurons, but can also be detected in other tissues such as human epidermal keratinocytes, the submandibular gland, and human hair follicles [12, 13]. Its activation can be triggered by various stimuli, such as heat >43°C, by protons, or by capsaicin and resiniferatoxin (RTX) [14, 15]. RTX is a chemical compound isolated from *Euphorbia resinifera* and *Euphorbia poissonii* which are cactus-like plants. RTX irreversibly binds TRPV1 and induces a sustained calcium influx [14, 16]. Additionally, RTX ablates TRPV1-expressing C-fibers, resulting in long-lasting pain relief [17, 18].

Previous studies suggest interactions between TNF and TRPV1, resulting in a modulation of ion channel function and protein expression in the pathophysiology of inflammatory and neuropathic pain [17, 19, 20]. TRPV1 was shown to be coexpressed with TNFR1 and with increased expression levels of TRPV1 by activating a TRPV1-dependent ERK signaling pathway in DRG neurons [21].

RTX was shown to enhance the frequency of spontaneous excitatory postsynaptic currents (EPSCs) between DRG neurons and dorsal horn neurons of the spinal cord. This results in the reduction of pain sensitization, because of TRPV1 overactivation, leading to receptor inhibition [17]. A decrease of TRPV1 protein level in the central nerve endings after intrathecal application of RTX was also observed, while TRPV1 protein level in peripheral nerve endings and DRG neurons was not affected [17]. In cultured DRG neuron somata, TNF sensitizes voltage-gated calcium channels (VGCC) against TRPV1 agonists [22, 23].

These data suggest a critical role for TRPV1 in TNF-induced neuropathic pain and an involvement of TNF in the potential analgesic effect of RTX.

In this study, we examine the interactions of TRPV1 and TNF for pain processing after intrathecal administration of RTX by using behavioral tests and detection of protein levels in DRG neurons of rats.

## 2. Materials and Methods

### 2.1. Animals

For behavioral experiments, male Sprague-Dawley (CD) rats were used (190–230 g, Charles River, Germany). All experiments were performed in accordance with the guidelines of the Animal Care and Use Committees of the University of Duisburg-Essen, Germany, and the German regulatory authorities (G939/07). All animals were kept on a 14/10 h light/dark cycle with water and standard food pellets available ad libitum.

### 2.2. Chemicals

TNF (Sigma-Aldrich, Germany) was dissolved in a mixture of bovine serum albumin (BSA, Sigma-Aldrich, Germany) and 0.9% sodium chloride (NaCl). TNF was used at concentrations of 2 ng/kg, 20 ng/kg, and 200 ng/kg. RTX (Alomone Labs, Israel) was dissolved in ethanol.

### 2.3. Drug Administration

Animals were anesthetized by using an isoflurane vaporizer with a gas flow of 500–1000 ml/min and 5% isoflurane concentration isoflurane. TNF and RTX were administered by intrathecal injections (10 *μ*l) as described previously [24]. Intrathecal injections were performed by inserting a 25 Ga × 1″ needle with a Hamilton syringe, into the tissues between the dorsal aspects L_5_ and L_6_, perpendicular to the spine. When the needle entered the subarachnoid space, a lateral tail movement could be observed, as an indicator of successful puncture. TNF was applied in concentrations of 2 ng/kg, 20 ng/kg, and 200 ng/kg per injection (adapted from [9]). RTX was administered in a concentration of 1.9 *μ*g/kg 60 minutes after TNF administration (adapted from [18]). All procedures were performed under aseptic conditions.

### 2.4. Behavioral Testing

Baseline data were recorded daily for 3 days. TNF, NaCl, RTX, or TNF/RTX was administered on day 4. Behavioral tests were performed 1, 3, 6, and 24 h after drug administration.

Thermal hyperalgesia was assessed with an algesimeter (Ugo Basile, Comerio, Italy), as described previously [25]. Three consecutive thermal tests were applied to the animals' hind paws with at least a 5 min interval between tests for an individual paw. Means of the three tests were calculated.

Mechanical sensitivity was assessed using von Frey hairs and the up-down method, as described elsewhere [26]. The 50% probability withdrawal threshold was determined. For each group, seven animals were tested (*n* = 7 for each condition; total of 56 animals).

### 2.5. Immunohistochemical Staining

DRG tissues were obtained as described previously [23]. Animals were sacrificed by overdose of isoflurane. The spinal column was removed and opened from the dorsal side. DRGs were carefully removed, postfixed in 4% paraformaldehyde for 24 h, frozen in liquid nitrogen, and stored until use at −80°C. Cryo-sections (8 *μ*m) of snap-frozen whole ganglia were placed onto microscopic slides. Three independent experiments were performed. DRGs of two animals per condition were pooled and randomly chosen for slice preparing. Five slices per condition were analyzed (*n* = 8 animals per experiment; total of 24 animals).

Tissue sections were fixed in 4% paraformaldehyde for 15 min and washed three times in phosphate-buffered saline (PBS). Cell membranes were permeabilized in PBS + 0.5% Triton X-100 for 15 min at room temperature. Unspecific binding sites were blocked with 5% milk powder in PBS for 1 h at room temperature with subsequent washing three times for 5 min at room temperature.

To investigate the relative protein level of TRPV1, slices were incubated with rabbit primary antibodies specific for TRPV1 receptor protein (1 : 500; Alomone Labs, Israel).

Mouse primary antibody for NeuN (1 : 500; Abcam) was used as a neuronal marker. Incubation in the primary antibody was performed for at 4°C overnight. After washing, the slices were incubated with secondary antibodies (goat anti-rabbit Alexa Fluor-488 or goat anti-mouse Cy3; 1 : 500; Dianova) for 1 h in the dark at room temperature.

### 2.6. Western Blot

For Western blot analysis, animals were treated as described above. DRG were removed 24 h after injection. The tissue was homogenized in RIPA buffer (Thermo Fisher Scientific) including protease inhibitor cocktail (Roche). Homogenates were subjected to 4–12% Bolt-Tris gels (Thermo Fisher Scientific). Proteins were transferred to nitrocellulose membranes (0.2 *μ*M) by using wet blotting technique. Membranes were blocked in Tris-buffered saline Tween-20 (TBST) containing 5% nonfat milk powder (Sigma-Aldrich) for 1 h at room temperature with gentle agitation. Membranes were incubated overnight at 4°C with rabbit polyclonal TRPV1 (1 : 200, Alomone Labs), rabbit polyclonal, and mouse polyclonal beta-III-tubulin antibodies (1 : 500, Sigma-Aldrich). Afterwards, membranes were incubated with anti-rabbit IgG and anti-mouse IgG horseradish peroxidase (1 : 1000, Thermo Fisher Scientific) for 2 h at room temperature. Immunoreactivity was detected by using enhanced chemiluminescence substrate (Thermo Fisher Scientific). For Western blot analysis, three independent approaches were performed with one animal per condition for each experiment (*n* = 12 animals in total).

### 2.7. Data Analysis

Data were analyzed using ANOVA and Tukey's post hoc test. Values have been checked for normal distribution using a Shapiro-Wilk normality test. A result was considered to be significant when *p* < 0.05 (^∗∗∗^*p* < 0.001; ^∗∗^*p* < 0.01; ^∗^*p* < 0.05). All data are reported as means ± SEM.

For examining alterations in TRPV1 protein level, DRG slices were analyzed with Zoe Fluorescent Cell Imager (Bio-Rad, Germany). Each image was taken with the same gain, brightness, and contrast values from all slices. TRPV1 protein levels were quantified using ImageJ software (NHI). Cells were selected using the Free Hand tool. The integrated density of TRPV1 in each NeuN-positive neuron was measured and normalized against the background signal in each image. Data were calculated from three independent approaches per condition. For each approach, a minimum of 100 cells from two rats were measured. Additional the number of TRPV1-positive neurons was counted and compared between each condition. Sensory neurons within the DRG were identified as TRPV1-positive when somata were clearly stained against NeuN (red) and TRPV1 (green), while TRPV1-negative neurons showed just a slight or no signal in the green channel.

Analysis of Western blot signals were performed by using ImageJ software (NIH). Intensity of TRPV1 were normalized to intensity of beta-III-tubulin as a neuronal marker.

## 3. Results

### 3.1. RTX Alleviates TNF-Induced Mechanical Allodynia

In von Frey testing, a concentration of 2 ng/kg or 200 ng/kg TNF led to a significant reduction of paw withdrawal threshold after 24 h compared to untreated control animals, resulting in an induction of mechanical allodynia. When TNF was administered in a concentration of 20 ng, no significant change in withdrawal threshold was observed in comparison to control animals, suggesting an inversed bell-shaped dose dependency.

**Figure 1:**
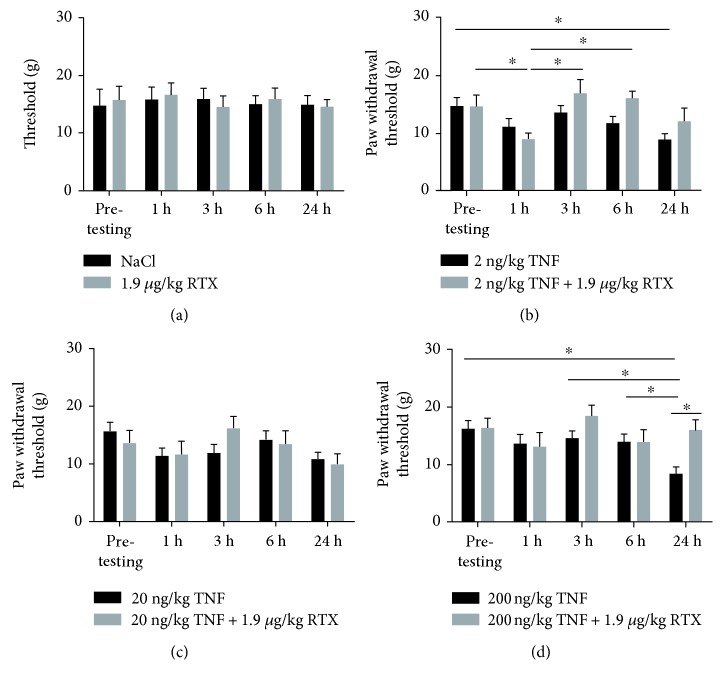
Influence of intrathecal administration of TNF and TNF + RTX on mechanical allodynia. (a) Administration of NaCl or RTX did not affect paw withdrawal thresholds, compared to control conditions (*p* > 0.05). (b) While 2 ng TNF does not influence paw withdrawal threshold 1, 3, or 6 h after administration, withdrawal threshold was reduced after 24 h (^∗^*p* < 0.05). Additional administration of RTX (1.9 mg/kg) did not abolish the TNF-induced reduction of withdrawal threshold 1 h, 3 h, 6 h, or 24 h after injection (*p* > 0.05). (c) Administration of TNF (20 ng) or the combination of TNF + RTX did not change paw withdrawal threshold (*p* > 0.05). (d) In contrast, administration of 200 ng TNF led to a reduction of paw withdrawal threshold after 24 h (^∗^*p* < 0.05). Additional administration of RTX abolished the effect of TNF after 24 h (^∗^*p* < 0.05).

When NaCl was administrated, no significant change in paw withdrawal threshold was observed, compared to untreated control animals (*p* > 0.05). When RTX was administrated solely, no change in behavior was observed (Figure 1(a)).

For examining the influence of 2 ng/kg TNF or the combined administration of 2 ng/kg TNF+ 1.9 mg/kg RTX, pretesting of untreated control animals showed a paw withdrawal threshold of 14.8 ± 1.4 g or 14.7 ± 1.9 g, respectively. When 2 ng TNF was administered, no significant change was observed after 1 (11.2 ± 1.4 g), 3 (13.7 ± 1.2 g), or 6 h (11.8 ± 1.1 g) compared to pretesting (*p* > 0.05). Twenty-four hours after TNF administration, paw withdrawal threshold was significantly decreased to 8.9 ± 1.1 g (*p* < 0.01). Additional administration of RTX resulted in a significant change of paw withdrawal threshold after 1 h (9.0 ± 1.0 g; *p* < 0.05), while after 3 (16.9 ± 2.3 g) or 6 h (16.1 ± 1.2 g), no significant change in paw withdrawal threshold was observed (*p* > 0.05), compared to pretests. Twenty-four hours after administration, paw withdrawal threshold was not significantly altered (12.1 ± 2.3 g) compared to the pretest (*p* < 0.05). There was no difference between TNF and TNF + RTX administration after 24 h (*p* > 0.05), but the additional administration of RTX increased of paw withdrawal threshold significantly 3 and 6 h after, compared to TNF administration (Figure 1(b)).

When TNF was administered in a concentration of 20 ng/kg or as a combination of 20 ng/kg TNF + RTX, no changes in paw withdrawal threshold were observed, compared to untreated control animals in pretests (Figure 1(c)).

In contrast to these findings, administration of 200 ng/kg TNF led to a reduction of paw withdrawal threshold after 24 h. During the pretesting of untreated animals, a withdrawal threshold of 16.2 ± 1.4 g was observed. Paw withdrawal threshold was reduced to 8.4 ± 1.2 g 24 h after TNF administration (*p* < 0.05). Additional administration of RTX did not change the paw withdrawal threshold over the time range, compared to pretesting (of 15.9 ± 1.9 g; *p* > 0.05). When comparing data of TNF and TNF + RTX, a significant difference was observed after 24 h. Paw withdrawal threshold was increased after additional RTX administration, compared to sole TNF administration (*p* < 0.05) (Figure 1(d)).

### 3.2. RTX Administration Alleviates TNF-Induced Heat Hyperalgesia

For examining heat hyperalgesia after intrathecal TNF administration, hot plate testing was performed. Only TNF in a concentration of 200 ng/kg led to a significant reduction of paw withdrawal threshold after 24 h compared to pretesting of untreated control animals. Administration of 2 or 20 ng/kg of TNF did not lead to significant change in paw withdrawal threshold over the experimental time.

**Figure 2:**
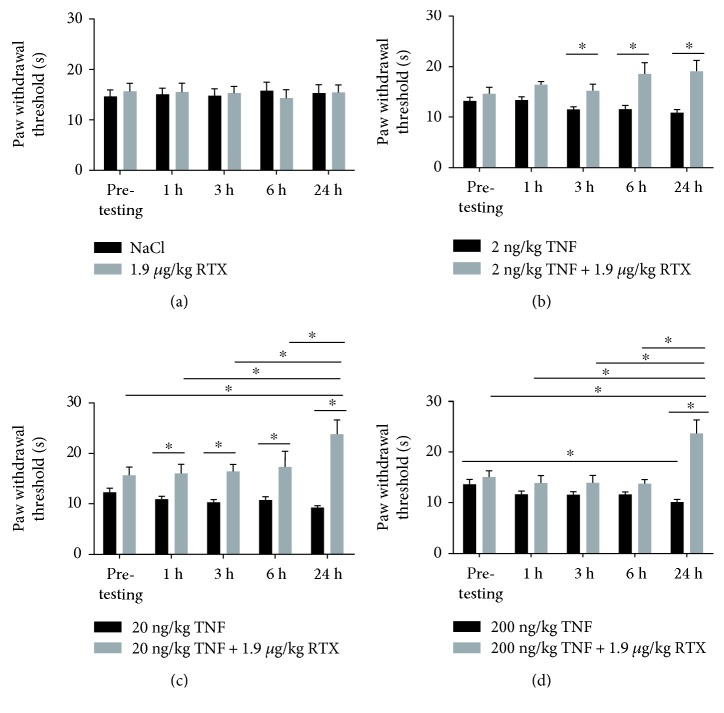
Influence of intrathecal administration of TNF and TNF + RTX on thermal hyperalgesia. (a) Administration of NaCl or RTX did not affect paw withdrawal thresholds, compared to control conditions (*p* > 0.05). (b) Two nanograms of TNF did not lead to changes in paw withdrawal thresholds 1 h, 3 h, 6 h, or 24 h after administration (*p* > 0.05). Additional administration of RTX (1.9 mg/kg) resulted in changes in withdrawal threshold over the experimental time. When TNF + RTX were administered, a significant increase of withdrawal threshold was observed after 3 (*p* < 0.05), 6 (*p* < 0.05), and 24 h (*p* < 0.05), compared to TNF administration alone. (c) Administration of TNF did not change paw withdrawal thresholds over the experimental time, compared to pretesting (*p* > 0.05). When RTX was additionally administered, paw withdrawal threshold was significantly increased (*p* < 0.05). When TNF + RTX was compared to TNF administration alone, an increase of withdrawal threshold was observed after 1 h (*p* < 0.05), 3 h (*p* < 0.05), 6 h (*p* < 0.05), and 24 h (*p* < 0.05). (d) Administration of 200 ng TNF resulted in a reduction of paw withdrawal threshold after 24 h (*p* < 0.05), while no changes were observed after 1 h, 3 h, or 6 h (*p* > 0.05). Additional RTX led to significant increase of paw withdrawal threshold 24 h after administration (*p* < 0.05). Additional RTX abolished the TNF-induced reduction of paw withdrawal threshold 24 h after administration (^∗^*p* < 0.05).

Administration of NaCl did not lead to significant alteration in paw withdrawal thresholds (*p* > 0.05). When RTX was administered to control animals, no change in behavior was observed (Figure 2(a)).

When examining the effect of 2 ng/kg TNF or 2 ng/kg TNF + RTX, pretesting of untreated animals resulted in a paw withdrawal threshold of 13.2 ± 0.7 g or 14.6 ± 1.3 g, respectively. When TNF was administered, no change in withdrawal threshold was observed after 1 h (13.4 ± 0.7 g), 3 h (11.5 ± 0.5 g), 6 h (11.6 ± 0.7 g), or 24 h (10.9 ± 0.6 g) compared to pretesting (*p* > 0.05). Additional administration of RTX resulted in changes in paw withdrawal threshold over the experimental time compared to pretesting. When TNF + RTX data were compared to TNF, a significant increase of paw withdrawal threshold could be observed 3 h (15.2 ± 1.2 g versus 11.5 ± 0.5 g; *p* < 0.05), 6 h (18.6 ± 2.1 g versus 11.58 ± 0.7 g; *p* < 0.05), and 24 h (19.1 ± 2.1 g versus 10.9 ± 0.6 g; *p* < 0.05) after administration (Figure 2(b)).

Administration of 20 ng/kg TNF or 20 ng/kg TNF + RTX resulted in a paw withdrawal threshold of 12.3 ± 0.8 g or 15.6 ± 1.6 g, respectively, during pretesting. Administration of TNF did not change paw withdrawal thresholds over the experimental time compared to pretesting (*p* > 0.05). Additional administration of RTX resulted in an increased paw withdrawal threshold of 23.8 ± 2.8 g after 24 h compared to pretesting (*p* < 0.05). When administration of TNF + RTX was compared to TNF administration alone, an increase of withdrawal threshold was observed after 1 h (16.0 ± 1.8 g versus 10.9 ± 0.5 g; *p* < 0.05), 3 h (16.4 ± 1.3 g versus 10.3 ± 0.4 g; *p* < 0.5), 6 h (17.3 ± 3.1 versus 10.7 ± 0.6 g; *p* < 0.05), and 24 h (23.8 ± 2.8 versus 9.2 ± 0.2 g; *p* < 0.05) (Figure 2(c)).

Pretesting for examining the influence of 200 ng/kg TNF or 200 ng/kg TNF + RTX resulted in paw withdrawal thresholds of 13.6 ± 0.9 g or 15.0 ± 1.2 g, respectively. Administration of TNF led to a reduction of paw withdrawal threshold to 10.1 ± 0.4 g after 24 h compared to pretesting. After 1 h (11.6 ± 0.6 g), 3 h (11.5 ± 0.6 g), or 6 h (11.6 ± 0.4 g), no changes in withdrawal thresholds were observed (*p* > 0.05). Additional administration of RTX led to a significant increase of paw withdrawal threshold after 24 h (23.7 ± 2.6 g), compared to pretesting (*p* < 0.05). Combined administration with RTX abolished the TNF-induced reduction of paw withdrawal threshold after 24 h (23.7 ± 2.6 g versus 10.1 ± 0.4 g; *p* < 0.05) (Figure 2(d)).

### 3.3. TNF Increases TRPV1 Protein Level after In Vivo Administration

**Figure 3:**
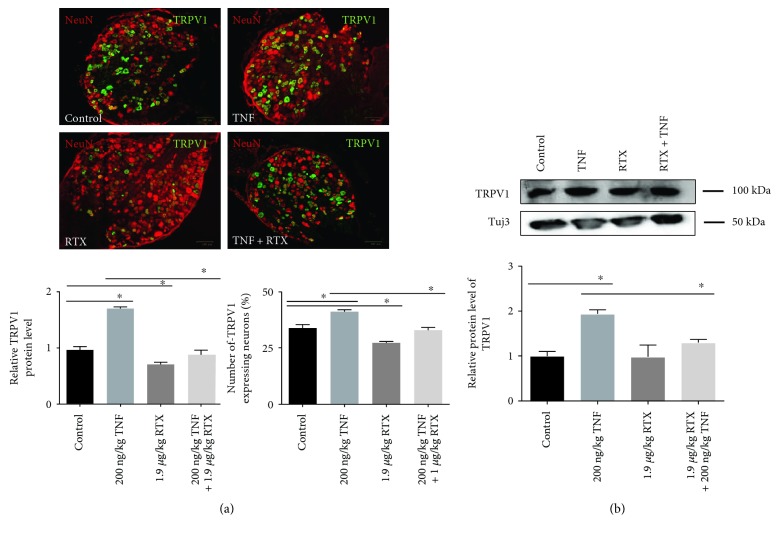
Influence of intrathecal administration of TNF and TNF + RTX on the protein level of TRPV1 and the number of TRPV1-expressing DRG neurons. (a) Immunohistochemical staining of DRG slices 24 h after administration of either 200 ng/kg TNF, 1.9 *μ*g/kg RTX, or 200 ng/kg TNF + 1.9 *μ*g/kg RTX. DRG neurons were stained against NeuN (red), as a neuronal marker and TRPV1 (green). Quantification of TRPV1-derived signals revealed significant increase in TRPV1 protein level after TNF administration, (*p* < 0.001), while administration RTX decreased the TRPV1 protein level significantly (*p* < 0.001), compared to control conditions. When TNF and TRPV1 were administrated together, a significant change to sole TNF administration was observed. Combined administration of TNF and RTX abolished the TNF-mediated increase of the TRPV1 protein level (*p* < 0.001). No significant change was observed when compared to untreated control (^∗^*p* > 0.05). In addition to the protein level of TRPV1, the number of TRPV1-expressing neurons within the DRG was significantly increased after TNF administration, compared to control conditions (^∗^*p* < 0.05). RTX administration significantly decreased the number of TRPV1-expressing neurons (^∗^*p* < 0.05). Combined application of TNF + RTX led to a significant reduction of TRPV1-positive neurons, compared to TNF administration (*p* < 0.01). When compared to control conditions, no significant alteration in the number of TRPV1-expressing neurons was observed (^∗^*p* > 0.05). (b) Western blot analysis showed an increase of TRPV1 protein level 24 h after TNF administration (^∗^*p* < 0.05), while additional administration of RTX reduced this effect (^∗^*p* < 0.05).

For examining the influence of TNF, RTX, or TNF + RTX on TRPV1 protein level, animals were treated with either TNF (200 ng/kg), RTX (1.9 *μ*g/kg), or a combination of TNF and RTX and sacrificed after 24 h. Protein levels of TRPV1 were determined in DRG slices by immunohistochemical staining (Figure 3(a)). In untreated control DRG neurons, a TRPV1 protein level of 1 ± 0.1 was measured. When animals were treated with TNF, TRPV1 protein level was significant increased to 1.77 ± 0.2 (*p* < 0.05), compared to control conditions. In vivo administration of RTX led to a significant decrease of the TRPV1 protein level after 24 h in comparison to untreated control DRGs (0.73 ± 0.1; *p* < 0.05). When RTX was administrated additionally to TNF, the TNF-induced increase of theTRPV1 protein level was abolished. Here, the protein level of TRPV1 was significantly decreased (0.91 ± 0.1; *p* < 0.05), compared to TNF administration additionally (Figure 3(a)).

When the protein level of TRPV1 was quantified with the Western blot technique, an increase of TRPV1 protein level after TNF (200 ng/kg) administration was observed (1.0 ± 0.1 versus 1.94 ± 0.09; *p* < 0.05). This effect was reduced by the additional administration of RTX (1.30 ± 0.06; *p* < 0.05) (Figure 3(b)).

### 3.4. TNF-Mediated Increase of TRPV1-Positive DRG Neurons Was Abolished by RTX

In addition to the influence of TNF and RTX on TRPV1 protein level, significant changes in the number of TRPV1-expressing neurons within the DRG were observed. While under control conditions, 33.8 ± 1.5% of DRG neurons were positive for TRPV1, and TNF led to an increased number of 41.2 ± 0.9% of TRPV1-expressing neurons (*p* < 0.05). In contrast, RTX administration significantly decreased the number of TRPV1-positive cells within the DRG to 27.3 ± 0.5% (*p* < 0.05), compared to control DRGs. When TNF and RTX were administrated simultaneously, RTX abolished the TNF-mediated increase of TRPV1-positive neurons (33 ± 1.2; *p* < 0.05) (Figure 3(a)).

## 4. Discussion

This study demonstrates the mechanism-based analgesic potential of intrathecal RTX administration on symptoms of TNF-induced pain, presenting as mechanical allodynia and thermal hyperalgesia in an inflammatory pain model. Additional intrathecal administration of the TRPV1 agonist RTX abolished TNF-induced thermal hyperalgesia and mechanical allodynia. These effects could be mediated by TNF-mediated increase and RTX-mediated decrease of TRPV1-expressing neurons within the DRG, underlining the critical involvement of TRPV1 in inflammatory and neuropathic pain.

TNF induces mechanical allodynia and thermal hyperalgesia 24 h after administration. This result is in concordance with recent studies showing similar results in rat and mice models of neuropathic pain [27–30]. The additional intrathecal administration of RTX inhibits the induction of both TNF-induced thermal hyperalgesia and mechanical allodynia which is consistent with the finding of a recent study [17] showing that intraplantar administration of carrageenan can induce thermal hyperalgesia. This effect was reduced by additional intrathecal administration of RTX (1.9 *μ*g/kg). In contrast, carrageenan-induced mechanical allodynia was not affected by RTX administration [18]. Capsaicin, another natural reversible-binding TRPV1 agonist, depletes substance P in primary sensory neurons after a single intrathecal injection resulting in a prolonged increase in the thermal, but not mechanical, pain thresholds [31, 32]. This contrasts with our results as we found an additional reduction of TNF-induced mechanical hyperalgesia after intrathecal administration of RTX. In other studies, the single intrathecal injection of RTX produced a rapid and significant increase in paw withdrawal latency to heat stimulation. Thus, capsaicin-sensitive unmyelinated afferents become desensitized or may degenerate after the treatment with RTX [33–36]. Furthermore, administration of RTX seems to be restricted to region of injection, while intrathecal administration of capsaicin can lead to a distribution throughout the cerebrospinal fluid (CSF); and therefore, effective concentrations may not have been achieved consistently [17]. In comparison to capsaicin, RTX is more selective in ablation of TRPV1 expression in nociceptors [17]. This may explain differences between both TRPV1 agonists.

TNF neutralizing antibodies were given intraoperatively after 4 days, in two different models of neuropathic pain, chronic constriction injury (CCI), and partial sciatic nerve transection (PST) [37]. Those findings are similar to our results, suggesting an influence of TNF on thermal hyperalgesia and mechanical allodynia and its reversible effect on antibodies or antagonists affecting TNF-induced pain. Nonetheless, clinical studies have failed. A cotherapy with antibodies specific for TNF and IL-1R1 reduced thermal hyperalgesia and mechanical allodynia more effectively than a monotherapy with single antibody [29]. Furthermore, TNFR1 is involved in pain behavior and neuronal activity in the absence of nerve injury, whereas TNFR2 may contribute in the presence of TNFR1 activation. It is suggested that TNFR2 is mostly found in small neurons, especially in coexpression with TRPV1. The similar reaction of wild-type and TRPV1-k.o. mice to mechanical stimuli suggests that TRPV1 is not involved in mediating mechanical sensitivity. These findings contrast with the observation that TRPV1 antagonists lead to a reduction of mechanical allodynia in models of neuropathic, inflammatory, or tumor pain, suggesting an additional role of TRPV1 in mechanical allodynia [38, 39]. Furthermore, these observations indicate possible, different mechanisms of mechanical sensitivity under physiological and pathophysiological conditions. TNF is known to increase the expression level of TRPV1 by activating TRPV1-dependent ERK signaling, but the influence of this overexpression on the effect of RTX is still unclear [40]. Here, we showed that the administration of TNF in vivo leads to an increase of the number of TRPV1-positive neurons in the DRG and to an increase of TRPV1 protein level, which is inconsistent with the findings of Hensellek et al. for the TNF treatment of cultured DRG neurons [21]. Furthermore, our results give evidence that this effect could be abolished by additional administration of RTX. While Jeffry and colleagues demonstrate a loss of TRPV1-expressing nerve terminals in the spinal cord, ten days after RTX administration, we could not confirm similar effects in the DRG after 24 h. Protein level and number of TRPV1-expressing neurons were significantly reduced but not eliminated [18]. This maybe be explained by the different investigated time points after RTX administration, so it could be possible that RTX also eliminates TRPV1 expression within the DRG after prolonged in exposition [17]. Nevertheless, we suggest RTX to mediate the reduction of TNF-induced thermal hyperalgesia by diminishing the enhanced TRPV1 protein level and the number of TRPV1-expressing neurons as well.

Additionally, electrophysiological studies suggest a higher analgesic potency of TRPV1 agonist in the presence of TNF. In this study, we provide evidence by observing the strongest induction of neuropathic symptoms and an analgesic effect of RTX 24 h after TNF or TNF + RTX administration during our behavioral experiments. This finding could be explained by TNF-induced overexpression of TRPV1 or by enhanced inhibition of VGCC [25].

## 5. Conclusions

Our results support a critical role of TRPV1 for typical signs of TNF-induced neuropathic pain, including thermal hyperalgesia and mechanical allodynia. Furthermore, we demonstrate an analgesic effect of TRPV1 agonist RTX, suggesting intrathecal TRPV1-acting drugs as a potential therapeutic approach for the treatment of neuropathic pain.
